# Antibiotics and Environment

**DOI:** 10.3390/antibiotics9040202

**Published:** 2020-04-23

**Authors:** Rosa Alduina

**Affiliations:** Department of Biological, Chemical and Pharmaceutical Sciences and Technologies (STEBICEF), University of Palermo, 90028 Palermo, Italy; valeria.alduina@unipa.it

Since the discovery of penicillin by Alexander Fleming in 1928, the use of antibiotics has become the golden standard in the treatment of bacterial infections of all kinds. Nowadays antibiotics are not only used in healthcare and in veterinary medicine but they are also employed in many human activities worldwide, such as fish farming, aquaculture, agriculture and in raising livestock. Therefore, wastewater from agriculture, hospitals, farms or urban wastewater treatment plants is increasingly likely to contain ranges of antibiotics, which eventually reach environmental niches with no previous history of contact to these compounds, for instance through water seepage or soil fertilisation. Because of their complex chemical structures, many antibiotics are resistant to degradation and, particularly in polluted areas, they can persist in the environment where they are thought to exert a positive selection pressure toward antibiotic-resistant bacteria. Thus, the environment represents a potential reservoir of antibiotics as well as antibiotic-resistant pathogens, which can then enter the food chain, leading to the selection of an antibiotic-resistant microbiota.

This Special Issue invited papers by experts working on antibiotic resistance in the environment in order to trace the prevalence of antibiotic-resistant bacteria or genes in wildlife and to propose suggestions to counteract the spread of these worrying pathogens. 

The study by Yoshizawa et al. [1] evaluated the ability of a tetracycline-resistant *Escherichia coli* strain and its *tetA* gene to survive in manure compost processing at different temperatures and additionally examined the incidence of oxytetracycline-resistant bacteria and the persistence of the *tetA* gene in Japanese pig and cow farms. Their findings demonstrated that both oxytetracycline-resistant *E. coli* and the resistance *tetA* gene persisted for many days in both pig and cow manure compost and that appropriate composting could decrease the concentration of tetracycline-resistant strains. The report by Rasschaert et al. [2] demonstrated that pig slurries contained traces of antibiotic and multiresistant *Salmonella* and *E. coli* strains, indicating that pig slurry used for fertilisation may be a source of soil contamination. High concentrations of antibiotic resistance genes and the presence of antibiotic-resistant bacteria in both manure compost and pig slurry may pose a risk to public health because these bacteria and genes could be transferred to natural soil microorganisms and then enter the human chain. Both these studies strongly advise for a controlled use of antimicrobials in raising livestock and in the treatment of manure compost.

Griffin et al. [3] found that antibiotic resistance genes were readily detectable in marine microbial communities proximal to a Florida Sewage Outfall System. The levels of antibiotic resistance genes showed seasonal differences, with a heightened prevalence in the wet season, potentially related to changes in seasonal water temperatures, variable seasonal wastewater discharge flows, seasonal antibiotic usage and coastal flow dynamics. Thus, the antibiotic resistance genes may present risks for recreational water use and to the coastal ecosystem itself. 

Antibiotic resistance is also related with environmental pollution and the presence of antibiotics and other pollutants in the coastal waters and rivers in China was summarised in the review by Xie et al. [4]. This study also suggests some strategies for future efforts in mitigating the phenomenon. 

While it is well-known that, even when used appropriately, antibiotics enhance selection for drug-resistant bacteria, the success of drug-resistant mutants is naturally mitigated by host immune response. The selective pressure applied by immune activation is lacking in immunocompromised hosts. This effect, coupled with the frequent antibiotic use of the immunocompromised patients, creates a novel evolutionary environment in which the selection for drug-resistant bacteria is drastically increased [5]. This is the case of infections due to *Mycobacterium tuberculosis* in immunocompromised HIV/AIDS populations. The study by DeNegre et al. [5] examined the evolutionary relationship between drug-sensitive and drug-resistant strains of *M. tuberculosis* and has direct implications for the communities of researchers studying the evolution and epidemiology of infectious diseases, especially in the context of diseases that compromise immunocompetence.

A few epidemiological studies have traced the prevalence of antibiotic-resistant bacteria in wildlife. Two reviews of this Issue focused on the molecular epidemiology and antibiotic resistance of *Staphylococcus aureus* lineages in wild animals worldwide [6] and in Europe [7], showing the distribution of molecular types, including those that are commonly found in humans or other animal species and represent a public health concern. *S. aureus* is a common bacterial colonizer of humans and a variety of animal species, and it is responsible for numerous types of infections. Many strains have zoonotic potential, i.e., the ability to cross interspecifically between humans and animals, such as livestock, pets, and wildlife. *S. aureus* is one of the bacterial species with a higher ability to acquire antibiotic resistance determinants and is usually associated with a multidrug-resistant profile. Antibiotic-resistant bacteria and antibiotic resistance genes were also described in marine environments, such as the Mediterranean Sea and its seacoast [8], and in wild marine animals, such as healthy sea turtles [9]. These studies confirmed that Gram-negative bacteria are most frequently found in samples deriving from the marine environment. Results obtained from sampling over several years suggest that the loggerhead sea turtle *Caretta caretta* may be a carrier of antibiotic resistance genes, probably due to its migratory habits and its longevity. 

Antibiotic resistance may be due to either genetic mutations or horizontal transfer of resistance genes, even among non-phylogenetically related bacteria. The acquisition of the antibiotic resistance genes is a natural phenomenon driven by evolution, but it can be accelerated by human activity. While many naturally occurring resistance genes are chromosomally located, antibiotic resistance genes are frequently associated with mobile genetic elements involved in the conjugative-mediated gene transfer commonly found in Gram-negative bacteria. The study by Sukmawinata et al. [10] demonstrated that the IncI1 plasmid was associated with the transmission of the gene *bla*_CTX-M-2_ in extended spectrum β-lactamase-producing *E. coli*, in thoroughbred racehorses in Japan. The study of Mohamed et al. [11] reported that conjugative Inc FII plasmids had an important role in the spread of carbapenemase-producing *Klebsiella pneumoniae* in Egypt. Conjugative plasmids play an important role in the spread of antibiotic resistance, and their recognition is essential to limit their diffusion. Integrons, together with plasmids, represent the mobile genetic elements that can mediate the transmission of antibiotic resistance genes via horizontal gene transfer mechanisms. A high percentage of the *int1* gene, encoding the mobile element class 1 integron, in environmental metagenomes was found [8] and this represents an alarming factor for the spreading of antibiotic resistance genes in the near future. The study by Liu et al. [12] described the occurrence of antibiotic resistance genes and mobile genetic elements known to enhance the spread of antibiotic resistance genes in two chicken farms where no antibiotics had been used for five years prior to the study. Their results provide a baseline for the occurrence of resistance genes in the chicken farms without direct selective pressure. 

All the studies above reported the contamination of environments with antibiotics and the presence of antibiotic-resistant bacterial strains and antibiotic resistance genes in every analysed sample. In addition, they suggest caution in the use of antibiotics and surveillance of resistant bacteria in every environment. Antibiotics are widely used today and will be so for the foreseeable future; new studies are therefore necessary to identify new activities for traditional molecules, to generate novel synthetic molecules for which bacteria have not developed any resistance, and to identify more potent and effective molecules. The review by Meade et al. [13] described bacteriocins, a class of non-toxic bacterial peptides that exhibit significant potency against certain bacteria (including multidrug-resistant species), and which could be considered as potential substitutes or aid to current therapeutic treatments. 

In our future search for new antibacterial activities, it will be difficult to obtain a silver bullet against pathogens without disturbing our microbiome. Thus, for all these reasons, the fight against bacterial pathogens is not over yet.
